# Native and non-native ambrosia beetles compete for deadwood resources

**DOI:** 10.1098/rspb.2026.1825

**Published:** 2026-09-16

**Authors:** Clayton R. Traylor, Michael D. Ulyshen, Scott Horn, David R. Coyle

**Affiliations:** ^1^ Department of Biology, Temple University, Philadelphia, PA, USA; ^2^ USDA Forest Service, Southern Research Station, Athens, GA, USA; ^3^ Department of Forestry and Environmental Conservation, Clemson University, Clemson, SC, USA

**Keywords:** invasive species impact, exploitative competition, saproxylic, priority effects

## Abstract

A consequence of biological invasion is competition between native and non-native species. Ambrosia beetles (Curculionidae: Scolytinae, Platypodinae) are among the most successful insect invaders, but their impact in natural settings is poorly understood. Across the United States, non-native ambrosia beetles numerically dominate their native counterparts, creating concern of competitive displacement. We compile one new and three published datasets to test for competition between native and non-native ambrosia beetles within their breeding resources. For each dataset, deadwood objects were created in the field, left to be naturally colonized by insects and then sampled for emergent insects using identical methods. Within 356 objects, native and non-native ambrosia beetles preferentially inhabited the same resources in space and time. Within 141 inhabited objects, native ambrosia beetle abundance declined with increasing abundance of non-natives (and vice versa). Furthermore, the probability of an emerging beetle being native was impacted by wood properties. Thus, we find that native and non-native ambrosia beetles compete, most likely in an exploitative manner, and that certain conditions provide competitive advantages for both groups. Given the importance of ambrosia beetles in forest ecosystem processes, further research is needed to understand the consequences of ambrosia beetle invasion in natural settings.

## 1. Introduction

Non-native species are increasingly common in natural settings around the world where they impact native species and ecosystem processes. Indeed, the impacts of invading species permeate into both aquatic and terrestrial ecosystems [1] and are observed across plants [2], vertebrates [3] and invertebrates [4]. Competition—when two or more species use the same limited space or resources, with one species’ usage resulting in less available for the other [5]—is one way that non-native species interact with native species [6]. Invaders often have traits or qualities that provide them with a competitive advantage over native species, such as rapid reproduction and growth [7], behavioural or phenotypic plasticity [8] and an absence of natural enemies [9]. Native species are thus negatively impacted by competition with functionally similar non-native species, but especially when invaders are abundant in the environment [10].

Some of the most successful insect invaders are ambrosia beetles (Coleoptera: Curculionidae: Scolytinae; Platypodinae). Spread among 16 weevil lineages, there are more than 3000 ambrosia beetle species worldwide, all of which have an obligate, symbiotic relationship with fungi [11–13]. Dispersing beetles transport their fungal mutualists within specialized organs called mycangia and then bore galleries into tree xylem within which they lay eggs and inoculate wood with their fungi. In turn, the fungi produce ambrosial mats that fully support the adult and larval nutrition; it is for this reason that ambrosia beetles are referred to as ‘fungus-farming’ beetles [14]. Ambrosia beetles are common invaders that are often introduced through infested wood products, wood-packaging material and live plants [15,16], but this is especially the case for Xyleborini whose haplodiploid life strategy and extremely polyphagous diet enable successful establishment and spread [17–19]. Since being introduced, some of these non-native species have become increasingly common in forest settings across wide geographic areas [20], where they outnumber native species many times over [21,22]. For example, just nine species of non-native ambrosia beetles accounted for 51% of >840 000 scolytines collected in a standardized, multi-year sampling effort across the United States [23]. This is not an isolated event: a meta-analysis of 39 studies found that non-native species represented on average 45% of Scolytinae abundance within a study, with values ranging from <1% to >96% [24]. Although there is uncertainty regarding the drivers of this variation and disproportionate attraction to trap lures used in these surveys [24,25], these examples nonetheless demonstrate that non-native ambrosia beetles are extremely abundant across a wide geographic area.

There are more than 50 ambrosia beetle species established outside of their native ranges [26]; however, only a few of these are known to be ecologically and economically important. Contrary to most native and non-native species that reproduce in deadwood, these species often attack living trees and can negatively impact agricultural, horticultural and forestry industries [14,27]. A few pests, such as the worldwide invaders *Xylosandrus crassiusculus* (Motschulsky) and *Xyleborinus saxesenii* (Ratzeburg), opportunistically attack stressed or weakened trees in great numbers, such as after spring flooding or frost damage [28,29]. These species do not cause widespread tree mortality in natural settings, but their damage results in large economic losses in orchards and plantations [30,31]. Others, such as *Xyleborus glabratus* Eichhoff and the *Euwallacea fornicatus* (Eichhoff) complex, attack living trees and have mutualist fungi that operate as plant pathogens [32,33]. Globally, widespread tree damage and mortality have resulted from their invasion in both natural and managed settings [34–36], resulting in cascading effects on other insects that rely on these tree species [37,38].

Ambrosia beetles play key roles in forest ecosystems [14]; thus, the superabundance of non-native species may impact ecosystem processes within the wood substrates they use and the organisms with whom they share these resources. Being some of the first colonizers of deadwood (including dying and stressed trees), the fungi that ambrosia beetles introduce have direct impact on wood decomposition [20,39]. Establishment of non-native ambrosia beetles may, therefore, accelerate or inhibit wood decay rates depending on the identity of their fungi. Furthermore, ‘priority effects’ imposed from ambrosia beetle galleries may influence the community trajectory of subsequent insect and fungi colonization [40,41]; but to our knowledge, these remain untested with ambrosia beetles and in relation to their invasion. Given the large abundance of a few non-native species, many have also speculated that non-native ambrosia beetles are competitively displacing native ambrosia beetles [21,23]. Indirect evidence of this, in the form of a meta-analysis of the relationship between native richness and non-native relative abundance for Scolytinae, is inconclusive [24]. Direct evidence of competition is absent.

Here, we compile new and published datasets to investigate competition between non-native and native ambrosia beetles within deadwood. To establish whether the potential for competition exists among ambrosia beetles, we first test if native and non-native species use the same deadwood resources (Hypothesis 1). After confirming their convergent resource use, we then test if the abundance of non-native ambrosia beetles negatively impacts native species (and *vice versa*; Hypothesis 2). Finally, we test for qualities of the deadwood objects that influence competitive outcomes between native and non-native ambrosia beetles (Hypothesis 3).

## 2. Methods

We compiled data from four deadwood emergence studies (one new and three published) to investigate competitive patterns of native and non-native ambrosia beetles within deadwood objects. As competition occurs when multiple individuals attempt to use the same finite resource [5], the spatial scale of our analysis (i.e. individual pieces of deadwood) is appropriate to examine competitive interactions. Moreover, our use of substrate sampling is preferred over trap sampling, which is the most common method for collecting and monitoring ambrosia beetles (e.g. [23]). Traps collect insects that emerge from a wide variety of deadwood substrates in a forest, meaning that interactions occurring at the scale of single deadwood objects are obscured [42,43].

All studies (table 1) were conducted in two areas of the southeastern United States, where many non-native ambrosia beetles are established and abundant in forest settings [44]. The studies involved creating fresh deadwood objects (logs or log bundles) in the field, allowing them to be colonized by insects, then bringing them into a rearing facility to collect emergent fauna. The tree species used (23 in total, including a mix of native/non-native species and pine/broadleaf species; supplementary material, table S1), length of time left in the field (1–13 months) and volume of deadwood objects (0.00375–0.086 m^3^) varied within and among studies. However, all studies employed an identical methodology in the same facility to sample insects inhabiting deadwood objects, as fully described in [45]. Note that only a single species of pine was used in these studies; thus, conclusions made about the type of tree (pine or broadleaf) are limited to this single tree species.

**Table 1.** Summary information for studies that supplied data in our analyses.

source	years sampled	ecoregion, state	total replicates (with any/native/non-native ambrosia beetles)	native abundance (% of total)	non-native abundance (% of total)
Ulyshen & Hanula [45]	2006–2007	Coastal Plain, South Carolina	144 (76/76/15)	11 043 (94.7%)	623 (5.3%)
Ulyshen *et al.* [46]	2011–2012	Piedmont, Georgia	48 (15/6/14)	60 (19.0%)	255 (81.0%)
Ulyshen *et al.* [47]	2018–2019	Coastal Plain, South Carolina	64 (9/5/4)	495 (65.3%)	263 (34.7%)
Present study	2021	Piedmont, Georgia	100 (41/19/37)	165 (7.3%)	2110 (92.7%)

*Notes:* Each study created deadwood objects, left them in the field to be colonized by insects and then used identical rearing methods to sample inhabiting insects. Replicates are single deadwood objects (or a bundle of objects) that are individually placed in rearing bags. Abundance is representative only of ambrosia beetles in the samples.

### (a) Overview of previously published data

Ulyshen & Hanula [45] created snags and logs of three native tree species (two broadleaf and one pine; supplementary material, table S1) at the Savannah River Site (Aiken and Barnwell counties), South Carolina (USA) on 5–6 June 2006 (table 1). The tree species included *Liquidambar styraciflua* L., *Quercus nigra* L. and *Pinus taeda* (L.). The tree species and postures (snag and log) were equally spread among two microclimates, with three replicates for each combination of species, posture and microclimate. The trees were left in the field for 11 months to be colonized by insects. In May 2007, they collected seven 0.5 m sections from each tree (one from the lower, middle and upper bole and three from the crown) and calculated the volume of each piece. Insects were sampled as stated below; each section of a bole was sampled individually, whereas the three crown sections from one tree were bundled together before sampling. In total, there were 144 sampled deadwood objects.

Ulyshen *et al*. [46] placed log sections of six tree species (all broadleaf; three native and three non-native; supplementary material, table S1) in forest settings at four sites in Clarke and Oconee counties, Georgia (USA) in May 2011. The native species included *Fraxinus pennsylvanica* Marshall, *L. styraciflua* and *Quercus phellos* L.; the non-native species included *Albizia julibrissin* Durazz., *Ligustrum sinense* Lour. and *Melia azedarach* L. The logs were cut into 0.5 m sections, with 10 logs of each species per site. After three months in the field (August 2011), they collected five logs per species from each site, bundled the logs of the same species together and began sampling insects as below. The volume of each log in a bundle was summed to provide a bundle volume. They repeated this process on the remaining logs after 13 months in the field (June 2012). In total, there were 48 deadwood objects (bundles) sampled.

Ulyshen *et al*. [47] set out log sections of two tree species (one native pine and one non-native broadleaf; supplementary material, table S1) in four forested blocks at the Savannah River Site in April 2018. The native pine was *P. taeda*; the non-native broadleaf was *Eucalyptus benthamii* Maiden & Cambage. Each block was composed of two plots, one within a monoculture of each tree species. Twelve logs of each species were placed in each plot, with four logs each representing one of three diameter groupings (approx. 6, 8 and 11 cm). After 1, 2, 6 and 12 months in the field, they returned to collect one log of each species and diameter group per plot. They bundled each plot's logs of the same species per time period for insect sampling, which followed as below. In total, there were 64 deadwood objects (bundles) sampled.

### (b) Collection of new data

In Clarke County, Georgia (USA), we set out experimental log bundles to be colonized by ambrosia beetles and subsequently sampled the emerging insects. A log bundle consisted of a small (2.5–5 cm diameter), medium (5–10 cm) and large (10–15 cm) log of a single tree zip-tied together (supplementary material, figure S1). All logs were 0.5 m in length, and we measured the diameter at the log's midpoint to calculate volume as a cylinder and then summed the three cylinders for bundle volume (m^3^). In total, there were 100 log bundles split among 20 broadleaf, deciduous tree species (supplementary material, table S1), with 5 replicates per species. Each replicate was from a unique tree. The tree species were chosen to be equally split between those native and non-native to Georgia, USA (*n* = 10 each), and for six of the non-native species, we included a closely related native (same genus or family; supplementary material, table S1). The other four non-native species had no native relatives, and so we chose other common native species. The native tree species included *Cercis canadensis* L., *F. pennsylvanica*, *L. styraciflua*, *Liriodendron tulipifera* L., *Morus rubra* L., *Q. nigra*, *Platanus occidentalis* L., *Prunus serotina* Ehrh., *Sassafras albidum* (Nutt.) Nees and *Ulmus alata* Michx. The non-native tree species included *Ailanthus altissima* (Mill.) Swingle, *A. julibrissin*, *Broussonetia papyrifera* (L.) Vent., *Camphora officinarum* Nees, *Firmiana simplex* (L.) W.Wight, *L. sinense*, *M. azedarach*, *Paulownia tomentosa* (Thunb.) Steud., *Pyrus calleryana* Decne. and *Ulmus parvifolia* Jacq.

All trees were cut down with permission from various locations in Clarke or Oconee County (Georgia) on 26 and 27 April 2021, except for *C. officinarum* which was sourced from Glynn County (coastal Georgia) on 22 April 2021. Log bundles were placed on the ground of a mature broadleaf forest within the Whitehall Experimental Forest (University of Georgia; 33.891° N, −83.365° E) on 27 April 2021. Bundles were aligned in a 10 × 10 grid with each separated by 2 m (supplementary material, figure S1); each bundle's position in the grid was randomized. Log bundles that were collected on days prior to the experimental start were temporarily housed indoors to prevent premature insect colonization. After six weeks, on 10 June 2021, we collected log bundles from the forest, brought them into our insect rearing facility and then followed the sampling methodology from [45]. Briefly, after collecting log bundles from the field, each bundle was hung from rafters with an eyehook drilled into the largest log, loosely wrapped inside a black trash bag and sealed with duct tape. At the bottom of the bag, we cut a hole and mounted a clear, plastic collection jar filled with propylene glycol to collect and preserve emergent insects (supplementary material, figure S1). To prevent mould growth from stagnant air in the bags, each wrapped bundle was also connected to a central air system via a tube that blew fresh air into the bag for one hour each day. The insect rearing facility has no heating or cooling systems, so the insects are exposed to ambient temperatures, and lights are kept on constantly to attract emergent insects to the collection jar. The bundles were monitored, and emergent insects were collected for over a year before logs were discarded. From the emergent insects, we identified beetles to species and calculated the number of native and non-native ambrosia beetles per wood bundle.

### (c) Data analysis

All statistical analyses were performed in R v.4.5.1 [48] using the glmmTMB package [49]. To test Hypothesis 1, we modelled the occurrence of native ambrosia beetles within all 356 deadwood objects, which we fit using a generalized linear mixed model (GLMM) with a binomial distribution (logistic link function). Independent variables included the presence/absence of non-native ambrosia beetles, the origin of the tree species (native or non-native), the type of tree (pine or broadleaf), wood volume (m^3^) and the length of time left in the field before sampling (months). We also included two random effects to account for study and the taxonomic family of the tree. This was the full model; we secondly fit a model that lacked a term for the presence/absence of non-native ambrosia beetles but was otherwise identical to the full model. These models were compared via AIC. To determine if the occurrence probability of non-native ambrosia beetles responded similarly to native species, we repeated the modelling process with the occurrence of non-native ambrosia beetles as the dependent variable and then used the presence/absence of native ambrosia beetles as an independent variable. In support of our hypothesis that native and non-native ambrosia beetles have similar substrate requirements and, therefore, co-occur within deadwood, we would expect the occurrence probability of native species to increase when objects are inhabited by non-native ambrosia beetles (and *vice versa*).

To test Hypothesis 2, we modelled the abundance of native ambrosia beetles using a negative binomial GLMM (log link function), using only deadwood objects in the dataset where ambrosia beetles occurred. Independent variables included the ln(1 + abundance) of non-native ambrosia beetles, origin of the tree species, type of tree and time left in the field before sampling. Tree family and study were included as random effects. In count models, an offset can be used to set expectations about counts owing to resource quantity and quality; the deviations from expectations are then related to independent variables. To isolate the effect of competition from non-native beetles on native abundance, we used offsets of wood volume as an expectation of resource quantity and total ambrosia beetle abundance per object as an expectation of resource quality (i.e. how productive each log was for all ambrosia beetles, regardless of origin). After controlling for how large and productive each log was for all ambrosia beetles, a negative relationship with non-native abundance would support our hypothesis and indicate that native species declined more than expected with increasing non-native abundance. We fit a second model that lacked a term for non-native ambrosia beetle abundance but was otherwise identical, and the two models were compared via AIC. To test if non-native species were reciprocally impacted by natives, we repeated this modelling process with non-native abundance as the response and ln(1+abundance) of native species as an independent variable.

To test Hypothesis 3, we modelled the probability of an emerging ambrosia beetle being native using a binomial GLMM (logistic link function), using only deadwood objects in the dataset where ambrosia beetles occurred. The probability of an outcome can be modelled with a binomial GLMM when the number of trials is known (in our case, the total abundance of ambrosia beetles per object). The dependent variable was thus specified as the number of successes (native beetles) and failures (non-native beetles) per deadwood object. Independent variables included the origin of the tree species, type of tree, wood volume and time left in the field before sampling; tree family and study were included as random effects. Significance of independent variables and predicted probabilities provide insight into which and how factors are driving competitive outcomes between native and non-native ambrosia beetles.

To verify the results and assess biases posed by particular studies, we repeated all portions of the analysis on four datasets that each excluded one of the studies listed in table 1. In one model of non-native occurrence excluding [47], we removed the fixed effect of tree type to allow for model convergence.

## 3. Results

### (a) Overview of the ambrosia beetle datasets

In the present study (new dataset), a total of 2275 individuals of nine ambrosia beetle species emerged from 41 of 100 deadwood objects. Six non-native species (all from Xyleborini) dominated abundance (92.7%), with the most abundant being *X. crassiusculus* (*n* = 1677 from 17 objects) and *X. saxesenii* (*n* = 356 from 10 objects). Other non-native species occurred in several objects but were low in abundance, such as *Xylosandrus germanus* (*n* = 34 from nine objects) and *Ambrosiodmus rubricollis* (*n* = 18 from 15 objects). The three native species from Corthylini and Xyleborini were all in relatively low abundance: *Xyleborus affinis* (*n* = 124 from 8 objects), *Xyleborus bispinatus* (*n* = 34 from 13 objects) and *Monarthrum mali* (*n* = 7 from two objects). Native and non-native ambrosia beetles emerged from 19 and 37 deadwood objects, respectively, including co-emergence from 15 objects. When non-native ambrosia beetles emerged, abundance ranged widely from 1 to 797 per object (mean ± SE = 57.0 ± 25.3). When native ambrosia beetles emerged, their abundance ranged from 1 to 109 (mean ± SE = 8.7 ± 5.6).

In the four studies combined, a total of 15 014 individuals of 19 ambrosia beetle species emerged (table 2), of which natives represented 11 763 individuals (78.3%) and non-natives represented 3251 individuals (21.7%). Ten native species were distributed among the Platypodini, Corthylini and Xyleborini, whereas nine non-native species were strictly Xyleborini (table 2). Out of 356 deadwood objects collected from the four studies, ambrosia beetles emerged from 141 (39.6%). Native and non-native ambrosia beetles emerged from 106 and 70 deadwood objects (29.8 and 19.7%), respectively, including co-emergence from 35 deadwood objects (9.8%). When native ambrosia beetles emerged, their abundance ranged widely from 1 to 700 per object (mean ± SE = 111.0 ± 15.8; supplementary material, figure S2). Similarly, when non-native ambrosia beetles emerged, their abundance ranged from 1 to 797 per object (mean ± SE = 46.4 ± 14.4; supplementary material, figure S2). There was considerable variation of these summary statistics among studies (table 1), which likely reflects idiosyncratic representation and abundance of species among studies (table 2; supplementary material, table S2). For each study, a breakdown of each species’ abundance per tree species is provided in tables S3–S6 in the supplementary material.

**Table 2.** Summary information for 19 ambrosia beetle species sampled in the four studies.

origin	tribe	species	number of studies present	proportion of objects occupied	abundance
native	Corthylini	*Gnathotrichus materiarius* (Fitch)	1	0.003	6
		*Monarthrum mali* (Fitch)	3	0.025	512
	Platypodini	*Euplatypus compositus* (Say)	1	0.098	3668
		*Myoplatypus flavicornis* (Fabricius)	1	0.014	242
		*Oxoplatypus quadridentatus* (Olivier)	1	0.081	3833
	Xyleborini	*Xyleborinus gracilis* (Eichhoff)	1	0.006	25
		*Xyleborus affinis* Eichhoff	3	0.115	2382
		*Xyleborus bispinatus* Eichhoff	2	0.039	39
		*Xyleborus ferrugineus* (Fabricius)	2	0.076	990
		*Xyleborus pubescens* Zimmermann	2	0.031	66
non-native	Xyleborini	*Ambrosiodmus rubricollis* (Eichhoff)	3	0.059	107
		*Ambrosiophilus atratus* (Eichhoff)	1	0.011	79
		*Cyclorhipidion bodoanum* (Reitter)	2	0.006	3
		*Dryoxylon onoharaense* (Murayama)	2	0.014	77
		*Euwallacea interjectus* (Blandford)	1	0.022	8
		*Xyleborinus saxesenii* (Ratzeburg)	4	0.084	1172
		*Xylosandrus compactus* (Eichhoff)	1	0.011	17
		*Xylosandrus crassiusculus* (Motschulsky)	3	0.065	1753
		*Xylosandrus germanus* (Blandford)	2	0.028	35

*Note:* A breakdown per study is provided in table S1 in the supplementary material, and species abundance per tree species is provided in tables S3–S6 in the supplementary material.

### (b) Hypothesis 1: use of the same deadwood resources

**Figure 1.**
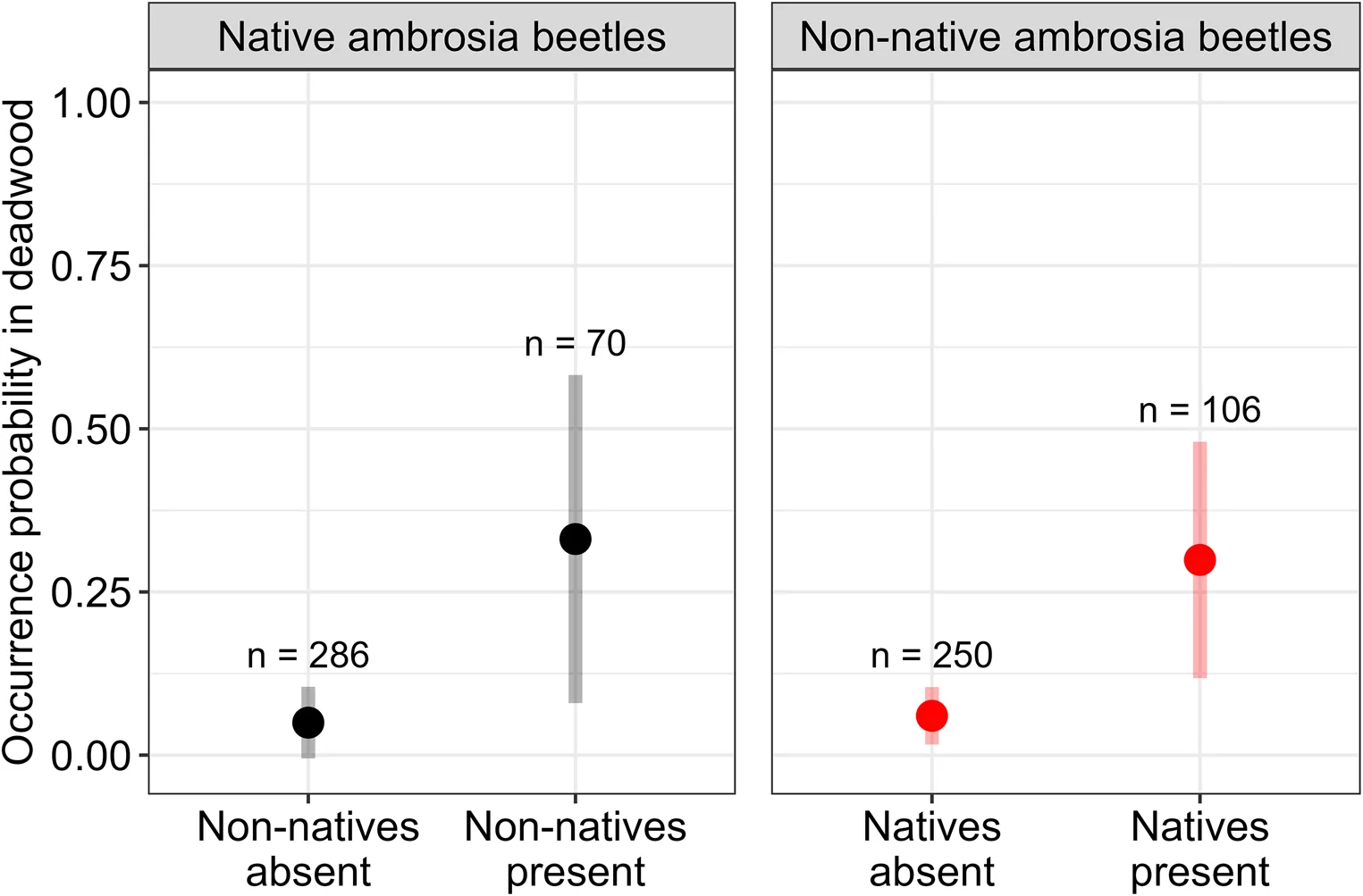
Predicted occurrence probability (mean ± SE) of native (left) and non-native (right) ambrosia beetles in deadwood objects increases when those objects are inhabited by the other.

In competing models of native ambrosia beetle occurrence in deadwood objects, the full model had a lower AIC by 26.7 than the model excluding the term for non-native presence (supplementary material, table S7). From the full model, the occurrence of native ambrosia beetles within deadwood objects was influenced by biotic and abiotic factors (supplementary material, table S8). First, the occurrence of native ambrosia beetles was higher in deadwood objects inhabited by non-native ambrosia beetles (*z*-value = 5.013, *p* = 5.358 × 10^−7^; figure 1). In deadwood objects where non-native ambrosia beetles were absent, the mean (±SE) occurrence probability of native ambrosia beetles was 0.050 (±0.055) but increased by 6.6 × to 0.331 (±0.251) when non-native ambrosia beetles were present (albeit with large uncertainty owing to a smaller sample size). Additionally, occurrence probability of native ambrosia beetles increased with the volume of deadwood objects (*z*-value = 4.955, *p* = 7.250 × 10^−7^) and declined with the number of months these objects were left in the field before sampling (*z*-value = −2.432, *p* = 0.015). However, native occurrence probability did not vary with tree origin or type (*z*-value ≤ 1.285, *p* ≥ 0.199).

Similarly, in competing models of non-native ambrosia beetle occurrence, the full model had a lower AIC by 18.7 than the model excluding the term for native presence (supplementary material, table S7). From the full model, occurrence of non-native ambrosia beetles was higher within deadwood objects inhabited by native ambrosia beetles (*z*-value = 4.224, *p* = 2.404 × 10^−5^; figure 1). In deadwood objects where native species were absent, the mean (±SE) occurrence probability of non-native ambrosia beetles was 0.060 (±0.044) but increased by 4.9 × to 0.299 (±0.181) when native ambrosia beetles were present. Tree type, tree origin, wood volume and time in the field did not influence the occurrence probability of non-native ambrosia beetles (|*z*-value| ≤ 1.106, *p* ≥ 0.269; supplementary material, table S8).

### (c) Hypothesis 2: patterns of abundance in used deadwood resources

**Figure 2.**
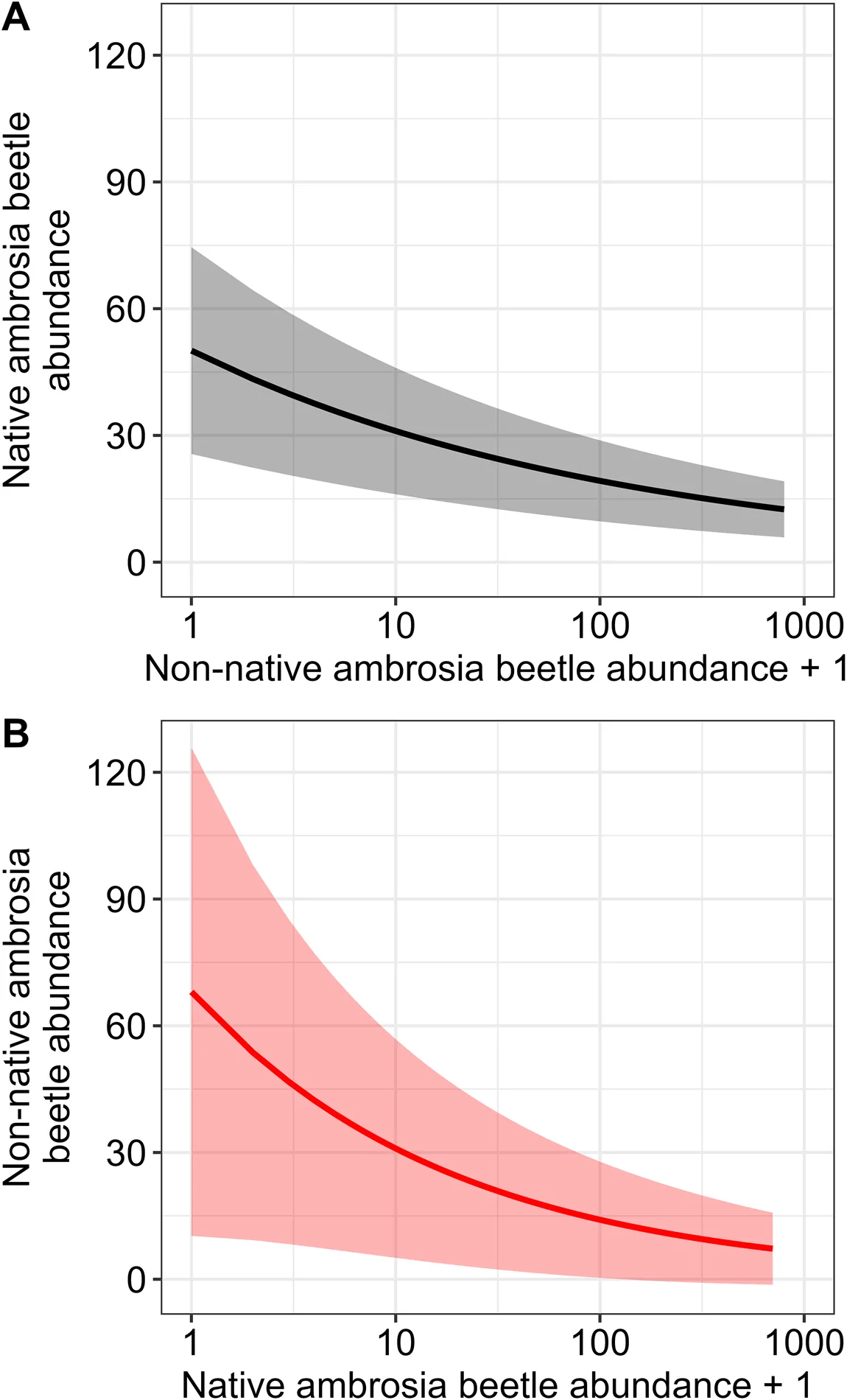
Patterns of competition between native and non-native ambrosia beetles within deadwood objects. Note the log scale on the *x*-axis. When accounting for the volume and total number of ambrosia beetles within deadwood objects, (A) abundance (mean ± SE) of native ambrosia beetles declines with increasing abundance of non-native species and (B) abundance (mean ± SE) of non-native ambrosia beetles declines with increasing abundance of native species.

In competing models of native ambrosia beetle abundance, the full model had a lower AIC by 15.4 than the model excluding the term for non-native abundance (supplementary material, table S7). After setting expectations from wood volume and productivity (i.e. total abundance) in the full model, the abundance of native ambrosia beetles declined with increasing abundance of non-native ambrosia beetles (*z*-value = −4.544, *p* = 5.525 × 10^−6^; figure 2A). The mean (±SE) abundance of native species decreased from 50.1 (±24.5) with 0 non-native beetles, to 34.5 (±16.6) with 5 non-native beetles, to 22.1 (±10.9) with 50 non-native beetles and to 13.8 (±7.2) with 500 non-native beetles. Native abundance was also higher in broadleaf than pine trees (*z*-value = 2.233, *p* = 0.026) and declined with increasing time that objects were left in the field (*z*-value = −2.685, *p* = 0.007). Native ambrosia beetle abundance did not vary with tree origin (*z*-value = 0.071, *p* = 0.943; supplementary material, table S8).

In competing models of non-native ambrosia beetle abundance, the full model had a lower AIC by only 1.9 than the model excluding the term for native abundance (supplementary material, table S7). After setting expectations for wood volume and productivity in the full model, non-native ambrosia beetle abundance declined with increasing abundance of native ambrosia beetles (*z*-value = −2.122, *p* = 0.034; figure 2B). The mean (±SE) abundance of non-native species decreased from 68.0 (±57.8) with 0 native beetles, to 36.9 (±30.4) with 5 native beetles, to 17.7 (±16.4) with 50 native beetles and to 8.1 (±9.2) with 500 native beetles. Non-native ambrosia beetle abundance was additionally higher in broadleaf than pine trees (*z*-value = 2.211, *p* = 0.027) but did not vary by tree origin or the length of time deadwood objects were left in the field (|*z*-value| ≤ 0.222, *p* ≥ 0.825; supplementary material, table S8).

### (d) Hypothesis 3: deadwood qualities influencing competitive outcomes

**Figure 3.**
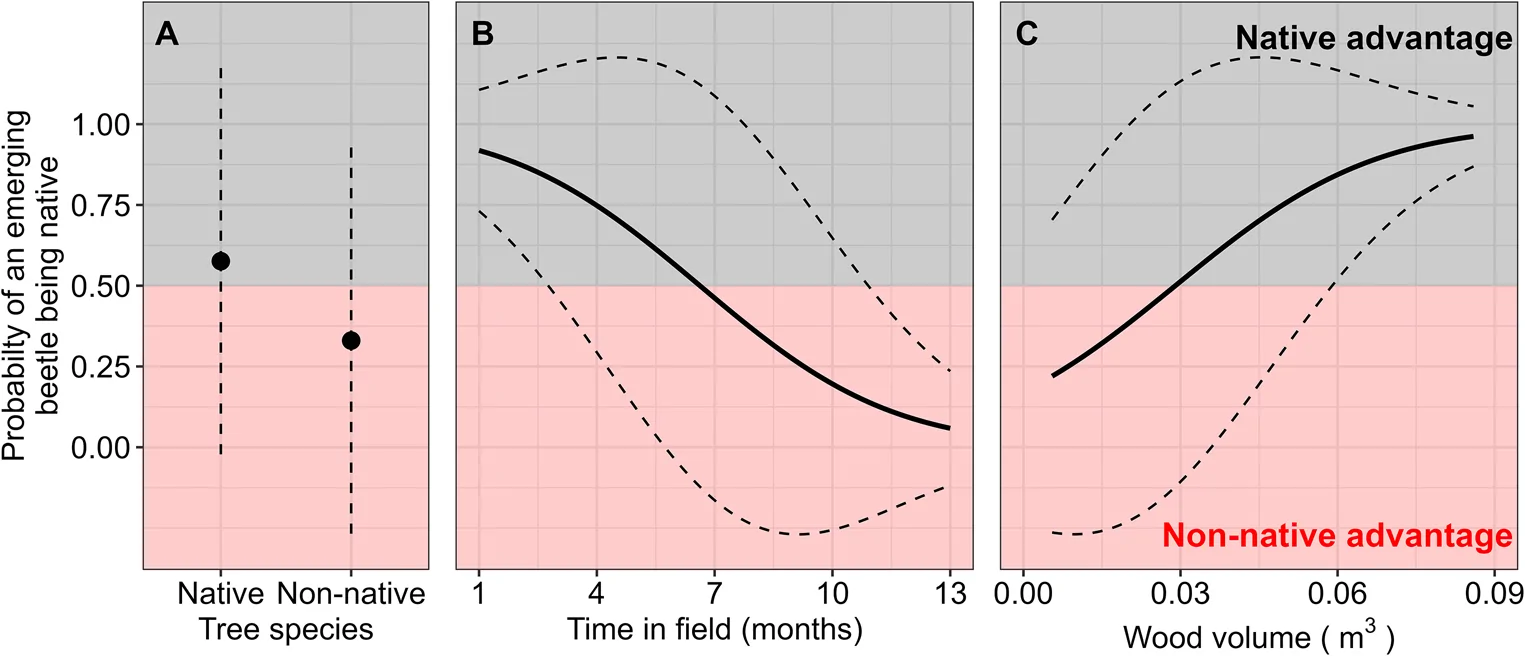
Patterns of competitive displacement of native and non-native ambrosia beetles within deadwood objects. The background of each panel is shaded to show when the probability of an emerging beetle being a native species is >0.5 (indicating native species advantage) versus <0.5 (indicating non-native species advantage). Solid points and lines show mean estimates, and dashed lines indicate ±SE. Probability of an emerging beetle being native (A) is higher in native than non-native tree species, (B) decreases with the length of time deadwood is left in the field before sampling and (C) increases with the volume of deadwood objects.

Three biotic and abiotic factors influenced the probability of an emerging beetle being native (supplementary material, table S8). First, the probability of an emerging beetle being native was higher in native than non-native tree species (*z*-value = 2.111, *p* = 0.035). The mean probability was 0.576 in native trees and 0.330 in non-native trees, indicating a slight advantage for native ambrosia beetles in native trees and non-native ambrosia beetles in non-native trees (figure 3A); however, large SE estimates (±0.625) demonstrate that these advantages are uncertain and probably weak. Second, the probability of an emerging beetle being native decreased with the length of time deadwood objects were left in the field before sampling (*z*-ratio = −12.221, *p* = 2.411 × 10^−34^). Mean estimates indicate a switch from native to non-native advantage after approximately 6.5 months, with SE estimates suggesting that advantages are weak for most of the spectrum and strongest at either extreme (figure 3B). Finally, the probability of an emerging beetle being native increased with the volume of the deadwood object (*z*-ratio = 15.217, *p* = 2.744 × 10^−52^). Mean estimates suggest a switch from non-native to native advantage at 0.03 m^3^; however, SE estimates suggest advantages are weak until volumes >0.06 m^3^ (figure 3C).

### (e) Validating results with dataset exclusions

The observed patterns of increased native ambrosia beetle occurrence when non-native species are present (and *vice versa*) and the decline of native abundance with increasing abundance of non-native species (and *vice versa*) were robust to excluding data from the individual studies in table 1 (supplementary material, tables S9–S12). The only exception was for non-native abundance, which no longer significantly declined with increasing native abundance when excluding [46]. Patterns of the probability of an emerging beetle being native were more sensitive to exclusion of individual studies (supplementary material, table S13).

## 4. Discussion

With the numerical dominance of non-native ambrosia beetles in trap samples across the United States, scientists have questioned if native species are being competitively displaced by non-natives [21,23,44]. Our study is the first to investigate the effects of non-native ambrosia beetles on native species at the resource scale (i.e. the spatial scale where competitive interactions take place). Competition occurs when species use the same finite resources, with one species’ usage depleting the quantity of resources available for the other [5]. In line with theory, we find that native ambrosia beetles are competitively displaced by their non-native counterparts. The two preferentially use the same deadwood objects, and in these objects, we find negative, density-dependent impacts of non-native ambrosia beetles on native species and *vice versa*. However, there is much to learn about the mechanisms of ambrosia beetle competition and the factors driving competitive outcomes between native and non-native species.

Our finding of native and non-native ambrosia beetles preferentially using the same deadwood pieces demonstrates their spatial and temporal overlap within finite resources (supporting Hypothesis 1), providing the basis for competitive interactions. These patterns are widely supported with anecdotal and experimental observations of ambrosia beetles establishing clustered galleries, often with several species present [20,28,29,50,51]. As wood resources vary considerably in chemical composition and structural attributes [52]—and as changes to these properties during decomposition make specific conditions (i.e. habitats) ephemeral and dynamic [53]—it is probable that constraints imposed by symbiotic fungi force multiple ambrosia beetle species to occupy the same wood pieces. For example, ambrosia beetles specialize on freshly killed or minimally decayed wood, including stressed trees and dying branches, with few exceptions (see [54]). These preferences provide optimal conditions for the growth of their saprotrophic mutualists that are only capable of consuming labile carbohydrates and not the structural components of wood [55]. As labile carbohydrates are depleted, the suitability of wood for the ambrosia beetle symbiosis quickly diminishes during decomposition [14]. Other conditions, such as elevated ethanol content in wood, may additionally benefit fungal mutualists by suppressing microbial antagonists and are commonly used by ambrosia beetles to detect suitable wood for gallery establishment [56,57]. At broader spatial scales, temperature and rainfall similarly drive native and non-native ambrosia beetle richness [44,58–60], providing further evidence of the functional overlap underpinning their competitive interactions.

Although inhabiting the same resources could indicate facilitation taking place, the negative impact of non-native abundance on native abundance (and native on non-native) solidifies that these interactions are competitive: there are negative and density-dependent effects of one group on the other (supporting Hypothesis 2). Considering the finite nature of suitable deadwood resources, we presume that increasing numbers of either native or non-native species limit space available for the other (i.e. exploitative competition [5]). This struggle for resources is often accompanied by interspecific hostility in other insects, particularly those with social behaviours [61,62]. However, aggressive behaviours have not been observed between ambrosia beetle species, at least in the only study on the topic [63]. It is possible that further research with a larger repertoire of species may reveal interspecific aggression, considering it is well known in the form of intraguild predation in other wood-boring beetles [64,65]. Niche partitioning is another way that phloem-feeding bark beetles avoid negative effects of competition [66,67]; in ambrosia beetles, niche partitioning of wood properties is likely driven by needs of the fungal symbiont [27]. However, as noted above, resource partitioning is not apparent in our study, at least at the scale of deadwood objects and with a non-taxonomic grouping by origin.

It is likely that subtle avoidance behaviours may alleviate negative consequences of ambrosia beetle competition. For example, Scabbio *et al*. [68] used X-ray tomography on ethanol-injected and flood-stressed trees to discover more than 12 700 intersections (>158 per 3.5 m tall tree) between the galleries of three ambrosia beetle species in Italy (*X. crassiusculus*, *X. germanus* and *X. saxesenii*). Among these, intraspecific intersections occurred more frequently than expected for all three species, whereas interspecific intersections occurred less frequently than expected [68]. These findings demonstrate that ambrosia beetles exhibit fine-scale aggregation behaviours to densely occupy resources with conspecifics but also employ fine-scale avoidance behaviours towards other species. They also complement reports of ambrosia beetles being attracted to volatiles of conspecific's ambrosial fungi [69,70] and preferring to establish galleries in wood already inhabited by conspecifics [51]. Aggregation behaviours such as these are likely to result in dense usage of wood resources. Conversely, ambrosia beetles are often repelled from the fungal volatiles of other ambrosia beetle genera [69] and ambrosia fungi compete for labile carbohydrates in wood [55]. Therefore, avoiding other species’ galleries may be a beetle's mechanism to promote the growth of its own fungal symbiont(s). It is likely that some ambrosia fungi are dominant over others, at least in certain abiotic conditions [27], which may also play into these dynamics. Certainly, more work is needed to understand patterns of competition among ambrosia beetles.

An important question concerns the factors driving competitive outcomes of native and non-native ambrosia beetles. Understanding what gives a competitive advantage to each group is essential for promoting native species with applied management and predicting other ecological impacts of ambrosia beetle invasion. We found that wood volume, length of time left in the field and tree species origin were related to the probability of an emerging beetle being native and thus likely contribute to competitive outcomes (supporting Hypothesis 3). Wood volume and length of time in the field may be explained by some natives specializing within larger deadwood pieces and some non-native species remaining later in the decomposition process. For example, *X. crassiusculus* and *X. saxesenii*, the most abundant non-natives in our study (table 2), are known to reproduce within a range of stem diameters greater than 1.5 cm [71]. On the other hand, the most common natives and some of the largest beetles in our study, *Euplatypus compositus* and *Oxoplatypus quadridentatus*, are restricted to larger diameter woods [72], and other abundant natives, such as *M. mali* and *Xyleborus ferrugineus*, prefer diameters of 10 cm or larger [71]. Furthermore, although most ambrosia beetles (native and non-native) specialize in fresh deadwood, *Ambrosiodmus* and *Ambrosiophilus* have wood-degrading fungal mutualists that allow them to remain in wood longer into the decomposition process [54,73]. Here, these genera were only represented by non-native species (table 2), which may explain the non-native advantage in older objects. Generally, these explanations are consistent with our observed patterns of occurrence: natives were selective in regard to length of time in the field and volume of the deadwood objects, whereas non-native species were not. Although the wood qualities noted above may determine the outcome of native and non-native ambrosia beetle competition in individual deadwood objects, all of these experimental conditions are found in virtually all forest settings. Thus, they fail to explain competitive displacement at larger scales.

One explanation for the spread and success of non-native ambrosia beetles may be the prevalence of non-native trees. For example, it has been previously posited that non-native insects benefit from both native and non-native plant diversity, whereas native insects only benefit from native plant diversity [74]. Indeed, invasive woody plants have become commonplace in forest ecosystems across the United States [75,76], and their ubiquity has facilitated the establishment and spread of many non-native insects [77,78]. Our results are generally in line with this: the probability of an emerging beetle being native was >0.5 in native trees and <0.5 in non-native trees, suggesting that native ambrosia beetles have a competitive advantage in native trees and non-native beetles in non-native trees. However, our analysis also reveals wide uncertainty in these estimates. Considering that tree origin was unimportant for the occurrence and abundance of both native and non-native ambrosia beetles (i.e. they are both using trees regardless of origin), non-native trees may not play a major role in ambrosia beetle invasion and competitive displacement. Indeed, host origin is thought to be unimportant for the fitness of highly polyphagous insects such as many ambrosia beetles [79].

Beyond wood conditions and qualities, competitive outcomes between native and non-native ambrosia beetles likely involve characteristics of the beetles themselves. For example, the success of the most prolific ambrosia beetle invaders (Xyleborini: *Euwallacea*, *Xylosandrus*, *Xyleborus*, *Xyleborinus*, etc.) is often attributed to host polyphagy and an inbreeding reproductive system that enables rapid reproduction even under low population densities [17–19]. These traits may also provide a competitive edge against other ambrosia beetles which have narrower host ranges and outbreeding systems. Indeed, high fecundity and niche plasticity are often attributed to the competitive success of invasive plants over native relatives [7,80]. Additionally, a competitive advantage may stem from the earlier and longer flight period of non-native ambrosia beetles [21]. While we did not have an appropriate dataset to test for this, the order of arrival is well known to influence competitive outcomes in other wood-boring insects and in ecological communities more generally [81,82]. Emerging earlier than natives gives non-native ambrosia beetles first access to stressed and dead trees from spring flooding or frost damage [28,29,83]. Moreover, it enables the symbiotic fungi of non-native species to establish before native competitors are searching for resources and thus may deter native species from forming galleries, as shown by Scabbio *et al*. [68]. Overall, these phenological differences, in combination with the overwhelming abundance of non-native species [21,23,84,85], may leave relatively few resources for native species to use. In a similar manner, the competitive success of invasive ants over natives has been partially attributed to simple numerical advantages [86]. We recommend future research to investigate this and other potential mechanisms that drive competitive displacement of native ambrosia beetles.

Relatively few ambrosia beetle species are among the world's most successful insect invaders and have well-documented and detrimental impacts on horticultural, agricultural and forestry industries [14,27]. It has been speculated that non-native ambrosia beetles may have impacts on native species as well, especially through competitive displacement of native ambrosia beetles [23]. Our study, for the first time, demonstrates that non-native ambrosia beetles competitively displace native species within deadwood resources, which are essential for their reproductive life cycle. As some of the earliest colonizers of deadwood, we may also expect ‘priority effects’ created by ambrosia beetles to impact the successional trajectory of later-arriving insect and fungal communities (e.g. [40,41]). Finally, ambrosia beetles play an important role in carbon cycling by introducing their fungi to deadwood; thus, their invasion may alter the rate of decomposition in forests [20,39,55]. Considering that non-native ambrosia beetles are numerically dominant in trap samples across the United States [23], it can be hypothesized that they place intense pressure on native ambrosia beetles and have great influence on ecosystem processes. However, these questions are largely untested at scale, emphasizing the urgent need to understand the ecological consequences of ambrosia beetle invasions.

## Supplementary Material



## Data Availability

The data and code used in this study are fully accessible in the following repository [87]. Supplementary material is available online [88].
