# Supplementary material for: Native and non-native ambrosia beetles compete for deadwood resources
Source: Proc Biol Sci. 2026 Sep 16;293(2079):20261825. doi: 10.1098/rspb.2026.1825 (PMC13577808; doi:10.1098/rspb.2026.1825)
Supplement: Supplementary file 1 [file rspb.2026.1825.zip › rspb.2026.1825/rspb20261825_si_001.docx]

Supplementary Electronic Material for:

**Native and non-native ambrosia beetles compete for deadwood resources**

Proceedings of the Royal Society B, 2026

DOI: <https://doi.org/10.1098/rspb.2026.1825>

Clayton R. Traylor^1^, Michael D. Ulyshen^2^, Scott Horn^2^, David R. Coyle^3^

^1^Department of Biology, Temple University, Philadelphia, Pennsylvania, USA

^2^Southern Research Station, U.S. Forest Service, Athens, Georgia, USA

^3^Department of Forestry and Environmental Conservation, Clemson University, Clemson, South Carolina, USA

Email: CRT (corresponding author; [clayton.r.traylor@gmail.com](mailto:clayton.r.traylor@gmail.com))

Figure S1. Methods for data collection in this study. A) Example of a deadwood bundle composed of a small (2.5–5 cm diameter), medium (5–10 cm), and large (10–15 cm) log; all logs were 0.5 m in length. B) Arrangement of 100 deadwood bundles on the forest floor in a 10×10 grid; each bundle was separated by 2 m and positioning in the grid was randomized. C) Bundles were collected from the field after 1 month and brought into the rearing facility to sample inhabiting insects. Each bundle was hung from rafters for the sampling protocol. D) Each bundle was wrapped in a black trash bag and equipped with a collection jar filled with propylene glycol at the bottom to collect emerging insects. A tube that delivered fresh air once per day is inserted in the bag at the top before sealing; tubes are more visible in panel C.

Figure S2. Abundance of native (y-axis) and non-native (x-axis) ambrosia beetles within deadwood objects. Each point represents a single deadwood object (points are transparent to allow visualization). Histograms on the plot exterior show the distribution of the raw data.

Table S1. List of native and non-native tree species in this analysis, and the number of replicates used in each study.

| Origin | Family | Species | Ulyshen and Hanula (2009) | Ulyshen et al. (2018) | Ulyshen et al. (2024) | Present study |
| --- | --- | --- | --- | --- | --- | --- |
| Non-native | Fabaceae | *Albizia julibrissin* Durazz. |  | 8 |  | 5 |
|  | Lauraceae | *Camphora officinarum* Nees |  |  |  | 5 |
|  | Malvaceae | *Firmiana simplex* (L.) W.Wight |  |  |  | 5 |
|  | Meliaceae | *Melia azedarach* L. |  | 8 |  | 5 |
|  | Moraceae | *Broussonetia papyrifera* (L.) Vent. |  |  |  | 5 |
|  | Myrtaceae | *Eucalyptus benthamii* Maiden & Cambage |  |  | 32 |  |
|  | Oleaceae | *Ligustrum sinense* Lour. |  | 8 |  | 5 |
|  | Paulowniaceae | *Paulownia tomentosa* (Thunb.) Steud. |  |  |  | 5 |
|  | Rosaceae | *Pyrus calleryana* Decne. |  |  |  | 5 |
|  | Simaroubaceae | *Ailanthus altissima* (Mill.) Swingle |  |  |  | 5 |
|  | Ulmaceae | *Ulmus parvifolia* Jacq. |  |  |  | 5 |
| Native | Altingiaceae | *Liquidambar styraciflua* L. | 48 | 8 |  | 5 |
|  | Fabaceae | *Cercis canadensis* L. |  |  |  | 5 |
|  | Fagaceae | *Quercus nigra* L. | 48 |  |  | 5 |
|  | Fagaceae | *Quercus phellos* L. |  | 8 |  |  |
|  | Lauraceae | *Sassafras albidum* (Nutt.) Nees |  |  |  | 5 |
|  | Magnoliaceae | *Liriodendron tulipifera* L. |  |  |  | 5 |
|  | Moraceae | *Morus rubra* L. |  |  |  | 5 |
|  | Oleaceae | *Fraxinus pennsylvanica* Marshall |  | 8 |  | 5 |
|  | Pinaceae | *Pinus taeda* (L.) | 48 |  | 32 |  |
|  | Platanaceae | *Platanus occidentalis* L. |  |  |  | 5 |
|  | Rosaceae | *Prunus serotina* Ehrh. |  |  |  | 5 |
|  | Ulmaceae | *Ulmus alata* Michx. |  |  |  | 5 |

Table S2. Summary information for 19 ambrosia beetle species sampled in the four studies. For each study, the species abundance (Abund) and proportion of deadwood objects occupied (Prop) is shown.

| Origin | Tribe | Species | Ulyshen and Hanula (2009) | | Ulyshen et al. (2018) | | Ulyshen et al. (2024) | | Present study | |
| --- | --- | --- | --- | --- | --- | --- | --- | --- | --- | --- |
|  |  |  | Abund | Prop | Abund | Prop | Abund | Prop | Abund | Prop |
| Native | Corthylini | *Gnathotrichus materiarius* (Fitch) | 6 | 0.007 | 0 | 0 | 0 | 0 | 0 | 0 |
|  |  | *Monarthrum mali* (Fitch) | 33 | 0.028 | 0 | 0 | 472 | 0.047 | 7 | 0.02 |
|  | Platypodini | *Euplatypus compositus* (Say) | 3,668 | 0.243 | 0 | 0 | 0 | 0 | 0 | 0 |
|  |  | *Myoplatypus flavicornis* (Fabricius) | 242 | 0.035 | 0 | 0 | 0 | 0 | 0 | 0 |
|  |  | *Oxoplatypus quadridentatus* (Olivier) | 3,833 | 0.201 | 0 | 0 | 0 | 0 | 0 | 0 |
|  | Xyleborini | *Xyleborinus gracilis* (Eichhoff) | 25 | 0.014 | 0 | 0 | 0 | 0 | 0 | 0 |
|  |  | *Xyleborus affinis* Eichhoff | 2,210 | 0.188 | 48 | 0.125 | 0 | 0 | 124 | 0.08 |
|  |  | *Xyleborus bispinatus* Eichhoff | 0 | 0 | 5 | 0.021 | 0 | 0 | 34 | 0.13 |
|  |  | *Xyleborus ferrugineus* (Fabricius) | 983 | 0.167 | 7 | 0.063 | 0 | 0 | 0 | 0 |
|  |  | *Xyleborus pubescens* Zimmermann | 43 | 0.063 | 0 | 0 | 23 | 0.031 | 0 | 0 |
| Non-native | Xyleborini | *Ambrosiodmus rubricollis* (Eichhoff) | 0 | 0 | 84 | 0.104 | 5 | 0.016 | 18 | 0.15 |
|  |  | *Ambrosiophilus atratus* (Eichhoff) | 0 | 0 | 79 | 0.083 | 0 | 0 | 0 | 0 |
|  |  | *Cyclorhipidion bodoanum* (Reitter) | 1 | 0.007 | 0 | 0 | 2 | 0.016 | 0 | 0 |
|  |  | *Dryoxylon onoharaense* (Murayama) | 56 | 0.028 | 0 | 0 | 21 | 0.016 | 0 | 0 |
|  |  | *Euwallacea interjectus* (Blandford) | 0 | 0 | 0 | 0 | 0 | 0 | 8 | 0.08 |
|  |  | *Xyleborinus saxesenii* (Ratzeburg) | 566 | 0.097 | 32 | 0.083 | 218 | 0.031 | 356 | 0.1 |
|  |  | *Xylosandrus compactus* (Eichhoff) | 0 | 0 | 0 | 0 | 0 | 0 | 17 | 0.04 |
|  |  | *Xylosandrus crassiusculus* (Motschulsky) | 0 | 0 | 59 | 0.104 | 17 | 0.016 | 1,677 | 0.17 |
|  |  | *Xylosandrus germanus* (Blandford) | 0 | 0 | 1 | 0.021 | 0 | 0 | 34 | 0.09 |

Table S3. Number of each ambrosia beetle species that emerged from deadwood objects of three tree species in Ulyshen and Hanula (2009).

| Beetle species | *Quercus nigra* | *Liquidambar styraciflua* | *Pinus taeda* |
| --- | --- | --- | --- |
| *Cyclorhipidion bodoanum* | 1 | 0 | 0 |
| *Dryoxylon onoharaense* | 0 | 56 | 0 |
| *Euplatypus compositus* | 1,829 | 1,839 | 0 |
| *Gnathotrichus materiarius* | 0 | 0 | 6 |
| *Monarthrum mali* | 18 | 15 | 0 |
| *Myoplatypus flavicornis* | 0 | 0 | 242 |
| *Oxoplatypus quadridentatus* | 3,794 | 39 | 0 |
| *Xyleborinus gracilis* | 25 | 0 | 0 |
| *Xyleborinus saxesenii* | 49 | 517 | 0 |
| *Xyleborus affinis* | 84 | 2,125 | 1 |
| *Xyleborus ferrugineus* | 20 | 179 | 784 |
| *Xyleborus pubescens* | 0 | 0 | 43 |

Table S4. Number of each ambrosia beetle species that emerged from deadwood objects of six tree species in Ulyshen et al. (2018).

| Beetle species | *Albizia julibrissin* | *Fraxinus pennsylvanica* | *Liquidambar styraciflua* | *Melia azedarach* | *Quercus phellos* | *Ligustrum sinense* |
| --- | --- | --- | --- | --- | --- | --- |
| *Ambrosiodmus rubricollis* | 78 | 0 | 5 | 1 | 0 | 0 |
| *Ambrosiophilus atratus* | 4 | 0 | 44 | 1 | 30 | 0 |
| *Xyleborinus saxesenii* | 0 | 0 | 14 | 0 | 0 | 18 |
| *Xyleborus affinis* | 17 | 0 | 20 | 0 | 11 | 0 |
| *Xyleborus bispinatus* | 0 | 0 | 0 | 0 | 5 | 0 |
| *Xyleborus ferrugineus* | 6 | 0 | 1 | 0 | 0 | 0 |
| *Xylosandrus crassiusculus* | 0 | 0 | 55 | 0 | 0 | 4 |
| *Xylosandrus germanus* | 1 | 0 | 0 | 0 | 0 | 0 |

Table S5. Number of each ambrosia beetle species that emerged from deadwood objects of two tree species in Ulyshen et al. (2024).

| Beetle species | *Pinus taeda* | *Eucalyptus benthamii* |
| --- | --- | --- |
| *Ambrosiodmus rubricollis* | 5 | 0 |
| *Cyclorhipidion bodoanum* | 0 | 2 |
| *Dryoxylon onoharaense* | 21 | 0 |
| *Monarthrum mali* | 0 | 472 |
| *Xyleborinus saxesenii* | 218 | 0 |
| *Xyleborus pubescens* | 13 | 10 |
| *Xylosandrus crassiusculus* | 0 | 17 |

Table S6. Number of each ambrosia beetle species that emerged from deadwood objects of 20 tree species in the present study.

| Beetle species | *Ailanthus altissima* | *Albizia julibrissin* | *Broussonetia papyrifera* | *Camphora officinarum* | *Cercis canadensis* | *Firmiana simplex* | *Fraxinus pennsylvanica* | *Ligustrum sinense* | *Liquidambar styraciflua* | *Liriodendron tulipifera* | *Melia azedarach* | *Morus rubra* | *Paulownia tomentosa* | *Platanus occidentalis* | *Prunus serotina* | *Pyrus calleryana* | *Quercus nigra* | *Sassafras albidum* | *Ulmus alata* | *Ulmus parvifolia* |
| --- | --- | --- | --- | --- | --- | --- | --- | --- | --- | --- | --- | --- | --- | --- | --- | --- | --- | --- | --- | --- |
| *Ambrosiodmus rubricollis* | 0 | 2 | 1 | 0 | 3 | 0 | 0 | 0 | 0 | 0 | 1 | 1 | 0 | 0 | 2 | 3 | 3 | 1 | 0 | 1 |
| *Euwallacea interjectus* | 0 | 1 | 0 | 0 | 1 | 2 | 0 | 0 | 0 | 0 | 3 | 0 | 0 | 0 | 0 | 0 | 1 | 0 | 0 | 0 |
| *Monarthrum mali* | 0 | 6 | 0 | 0 | 0 | 0 | 0 | 0 | 0 | 0 | 1 | 0 | 0 | 0 | 0 | 0 | 0 | 0 | 0 | 0 |
| *Xyleborinus saxesenii* | 0 | 19 | 0 | 0 | 3 | 0 | 0 | 1 | 331 | 0 | 0 | 0 | 0 | 0 | 2 | 0 | 0 | 0 | 0 | 0 |
| *Xyleborus affinis* | 1 | 116 | 0 | 0 | 2 | 0 | 0 | 0 | 3 | 0 | 0 | 0 | 0 | 0 | 2 | 0 | 0 | 0 | 0 | 0 |
| *Xyleborus bispinatus* | 0 | 21 | 0 | 0 | 0 | 0 | 1 | 0 | 1 | 0 | 0 | 3 | 0 | 0 | 0 | 2 | 4 | 2 | 0 | 0 |
| *Xylosandrus compactus* | 0 | 1 | 0 | 14 | 1 | 0 | 0 | 1 | 0 | 0 | 0 | 0 | 0 | 0 | 0 | 0 | 0 | 0 | 0 | 0 |
| *Xylosandrus crassiusculus* | 2 | 1,004 | 0 | 0 | 1 | 0 | 0 | 6 | 636 | 0 | 22 | 4 | 0 | 0 | 2 | 0 | 0 | 0 | 0 | 0 |
| *Xylosandrus germanus* | 1 | 29 | 0 | 0 | 0 | 0 | 0 | 1 | 2 | 0 | 1 | 0 | 0 | 0 | 0 | 0 | 0 | 0 | 0 | 0 |

Table S7. Model comparisons for four GLMMs. For each response, a full model was compared with AIC to an identical model where an independent variable was excluded. The lowest AIC value for each comparison is bold and the differences between models (ΔAIC) is shown.

| Response variable | Independent variable excluded | df (full) | df (excluded) | AIC (full) | AIC (excluded) | ΔAIC |
| --- | --- | --- | --- | --- | --- | --- |
| Native occurrence | Non-native presence/absence | 8 | 7 | **314.2** | 340.9 | -26.7 |
| Non-native occurrence | Native presence/absence | 8 | 7 | **282.6** | 301.3 | -18.7 |
| Native abundance | ln(1+non-native abundance) | 8 | 7 | **1007.6** | 1023.0 | -15.4 |
| Non-native abundance | ln(1+native abundance) | 8 | 7 | **664.2** | 666.1 | -1.9 |

Table S8. Model summaries for generalized linear models of ambrosia beetle occurrence, abundance, and probability within deadwood objects. Each model has random effects for study and tree family. Occurrence models include all 356 deadwood objects; abundance and probability models include only the 141 deadwood objects inhabited by ambrosia beetles. Abundance models have offsets for deadwood volume and total number of ambrosia beetles to set count expectations for resource quantity and quality. Z-values in bold are significant (*p*-value < 0.05).

| Response | Variables | Estimate | SE | z-value | p-value |
| --- | --- | --- | --- | --- | --- |
| Native occurrence | (Intercept) | -3.435 | 1.431 | **-2.400** | 0.016 |
|  | Non-native ambrosia present | 2.335 | 0.466 | **5.013** | 5.36×10^-07^ |
|  | Origin (native) | 0.669 | 0.521 | 1.285 | 0.199 |
|  | Type (broadleaf) | 0.922 | 0.797 | 1.157 | 0.247 |
|  | Wood volume (m^3^) | 65.502 | 13.221 | **4.955** | 7.25×10^-07^ |
|  | Time (months) | -0.263 | 0.108 | **-2.432** | 0.015 |
| Non-native occurrence | (Intercept) | -2.898 | 1.480 | **-1.959** | 0.050 |
|  | Native ambrosia present | 2.017 | 0.478 | **4.224** | 2.40×10^-05^ |
|  | Origin (native) | -0.522 | 0.488 | -1.070 | 0.285 |
|  | Type (broadleaf) | 1.403 | 1.373 | 1.022 | 0.307 |
|  | Wood volume (m3) | 1.556 | 14.297 | 0.109 | 0.913 |
|  | Time (months) | -0.082 | 0.074 | -1.106 | 0.269 |
| Native abundance | (Intercept) | 3.626 | 0.532 | **6.815** | 9.43×10^-12^ |
|  | ln(1 + non-native abundance) | -0.208 | 0.046 | **-4.544** | 5.52×10^-06^ |
|  | Origin (native) | 0.021 | 0.290 | 0.071 | 0.943 |
|  | Type (broadleaf) | 0.443 | 0.198 | **2.233** | 0.026 |
|  | Time (months) | -0.136 | 0.051 | **-2.685** | 0.007 |
| Non-native abundance | (Intercept) | 1.694 | 1.520 | 1.114 | 0.265 |
|  | ln(1 + native abundance) | -0.342 | 0.161 | **-2.122** | 0.034 |
|  | Origin (native) | 0.028 | 0.537 | 0.051 | 0.959 |
|  | Type (broadleaf) | 2.294 | 1.037 | **2.211** | 0.027 |
|  | Time (months) | -0.026 | 0.115 | -0.222 | 0.825 |
| Probability of an emerging beetle being native | (Intercept) | 1.795 | 3.633 | 0.494 | 0.621 |
|  | Origin (native) | 1.148 | 0.544 | **2.111** | 0.035 |
|  | Type (broadleaf) | -1.456 | 3.074 | -0.474 | 0.636 |
|  | Wood volume (m^3^) | 63.389 | 4.166 | **15.217** | 2.74×10^-52^ |
|  | Time (months) | -0.491 | 0.040 | **-12.221** | 2.41×10^-34^ |

Table S9. Model summaries for generalized linear models of native ambrosia beetle occurrence within deadwood objects. Each model excludes a portion of the dataset originating from a unique study (Table 1 in the Main Text) and had random effects for study and tree family. Z-values in bold are significant (*p*-value < 0.05).

| Response: Native ambrosia beetle occurrence | | | | | |
| --- | --- | --- | --- | --- | --- |
| Excluded study | Variables | Estimate | SE | z-value | p-value |
| Excluding Ulyshen and Hanula (2009) | (Intercept) | -3.627 | 1.392 | **-2.606** | 0.009 |
|  | Non-native ambrosia present | 1.895 | 0.497 | **3.812** | 1.38×10^-04^ |
|  | Origin (native) | 0.515 | 0.524 | 0.982 | 0.326 |
|  | Type (broadleaf) | 0.913 | 1.338 | 0.683 | 0.495 |
|  | Wood volume (m^3^) | 32.077 | 21.963 | 1.461 | 0.144 |
|  | Time (months) | -0.306 | 0.134 | **-2.278** | 0.023 |
| Excluding Ulyshen et al. (2018) | (Intercept) | -4.852 | 0.629 | **-7.718** | 1.19×10^-14^ |
|  | Non-native ambrosia present | 2.016 | 0.440 | **4.583** | 4.59×10^-06^ |
|  | Origin (native) | 0.807 | 0.432 | 1.869 | 0.062 |
|  | Type (broadleaf) | 1.305 | 0.408 | **3.198** | 0.001 |
|  | Wood volume (m^3^) | 79.018 | 14.078 | **5.613** | 1.99×10^-08^ |
|  | Time (months) | 0.096 | 0.046 | **2.113** | 0.035 |
| Excluding Ulyshen et al. (2024) | (Intercept) | -3.807 | 1.720 | **-2.214** | 0.027 |
|  | Non-native ambrosia present | 2.819 | 0.557 | **5.063** | 4.12×10^-07^ |
|  | Origin (native) | 0.971 | 0.584 | 1.663 | 0.096 |
|  | Type (broadleaf) | 0.673 | 0.840 | 0.801 | 0.423 |
|  | Wood volume (m^3^) | 62.631 | 13.181 | **4.752** | 2.02×10^-06^ |
|  | Time (months) | -0.220 | 0.116 | -1.890 | 0.059 |
| Excluding present study | (Intercept) | -3.042 | 1.735 | -1.753 | 0.080 |
|  | Non-native ambrosia present | 2.500 | 0.661 | **3.784** | 1.54×10^-04^ |
|  | Origin (native) | 0.714 | 0.805 | 0.886 | 0.376 |
|  | Type (broadleaf) | 1.109 | 0.608 | 1.824 | 0.068 |
|  | Wood volume (m^3^) | 64.339 | 13.218 | **4.868** | 1.13×10^-06^ |
|  | Time (months) | -0.285 | 0.116 | **-2.451** | 0.014 |

Table S10. Model summaries for generalized linear models of non-native ambrosia beetle occurrence within deadwood objects. Each model excludes a portion of the dataset originating from a unique study (Table 1 in the Main Text) and had random effects for study and tree family. Z-values in bold are significant (*p*-value < 0.05).

| Response: Non-native ambrosia beetle occurrence | | | | | |
| --- | --- | --- | --- | --- | --- |
| Excluded study | Variables | Estimate | SE | z-value | p-value |
| Excluding Ulyshen and Hanula (2009) | (Intercept) | -2.280 | 1.411 | -1.616 | 0.106 |
|  | Native ambrosia present | 1.776 | 0.534 | **3.326** | 0.001 |
|  | Origin (native) | -0.400 | 0.491 | -0.815 | 0.415 |
|  | Type (broadleaf) | 1.407 | 1.390 | 1.012 | 0.311 |
|  | Wood volume (m^3^) | -8.344 | 18.461 | -0.452 | 0.651 |
|  | Time (months) | -0.047 | 0.064 | -0.738 | 0.460 |
| Excluding Ulyshen et al. (2018) | (Intercept) | -2.416 | 1.504 | -1.607 | 0.108 |
|  | Native ambrosia present | 1.925 | 0.489 | **3.939** | 8.17×10^-05^ |
|  | Origin (native) | -0.206 | 0.562 | -0.367 | 0.714 |
|  | Type (broadleaf) | 1.593 | 1.457 | 1.094 | 0.274 |
|  | Wood volume (m^3^) | 6.099 | 17.056 | 0.358 | 0.721 |
|  | Time (months) | -0.289 | 0.076 | **-3.827** | 1.29×10^-04^ |
| Excluding Ulyshen et al. (2024) | (Intercept) | -1.784 | 1.087 | -1.642 | 0.101 |
|  | Native ambrosia present | 2.596 | 0.570 | **4.555** | 0.000 |
|  | Origin (native) | -0.849 | 0.522 | -1.627 | 0.104 |
|  | Wood volume (m^3^) | -0.385 | 15.594 | -0.025 | 0.980 |
|  | Time (months) | -0.047 | 0.081 | -0.578 | 0.563 |
| Excluding present study | (Intercept) | -3.193 | 1.719 | -1.857 | 0.063 |
|  | Native ambrosia present | 2.140 | 0.647 | **3.305** | 0.001 |
|  | Origin (native) | -0.867 | 0.993 | -0.873 | 0.383 |
|  | Type (broadleaf) | 1.291 | 1.418 | 0.910 | 0.363 |
|  | Wood volume (m^3^) | 5.744 | 15.338 | 0.374 | 0.708 |
|  | Time (months) | -0.045 | 0.069 | -0.647 | 0.517 |

Table S11. Model summaries for generalized linear models of native ambrosia beetle abundance within deadwood objects. Each model excludes a portion of the dataset originating from a unique study (Table 1 in the Main Text), had random effects for study and tree family, and had offsets for wood volume and total number of ambrosia beetles in the object. Z-values in bold are significant (*p*-value < 0.05).

| Response: Native ambrosia beetle abundance | | | | | |
| --- | --- | --- | --- | --- | --- |
| Excluded study | Variables | Estimate | SE | z-value | p-value |
| Excluding Ulyshen and Hanula (2009) | (Intercept) | 3.825 | 1.067 | **3.585** | 3.37×10^-04^ |
|  | log(1 + Non-native abundance) | -0.419 | 0.106 | **-3.944** | 8.01×10^-05^ |
|  | Origin (native) | 0.190 | 0.520 | 0.366 | 0.714 |
|  | Type (broadleaf) | -0.002 | 1.028 | -0.002 | 0.998 |
|  | Time (months) | -0.202 | 0.061 | **-3.289** | 0.001 |
| Excluding Ulyshen et al. (2018) | (Intercept) | 6.781 | 2.493 | **2.720** | 0.007 |
|  | log(1 + Non-native abundance) | -0.163 | 0.044 | **-3.725** | 1.96×10^-04^ |
|  | Origin (native) | -0.091 | 0.339 | -0.269 | 0.788 |
|  | Type (broadleaf) | 0.383 | 0.176 | **2.170** | 0.030 |
|  | Time (months) | -0.764 | 0.304 | **-2.517** | 0.012 |
| Excluding Ulyshen et al. (2024) | (Intercept) | 2.929 | 0.925 | **3.166** | 0.002 |
|  | log(1 + Non-native abundance) | -0.166 | 0.049 | **-3.367** | 0.001 |
|  | Origin (native) | 0.336 | 0.503 | 0.668 | 0.504 |
|  | Type (broadleaf) | 0.510 | 0.477 | 1.070 | 0.285 |
|  | Time (months) | -0.110 | 0.053 | **-2.086** | 0.037 |
| Excluding present study | (Intercept) | 3.943 | 0.660 | **5.975** | 2.30×10^-09^ |
|  | log(1 + Non-native abundance) | -0.141 | 0.048 | **-2.915** | 0.004 |
|  | Origin (native) | 0.028 | 0.371 | 0.075 | 0.940 |
|  | Type (broadleaf) | 0.404 | 0.174 | **2.327** | 0.020 |
|  | Time (months) | -0.163 | 0.047 | **-3.436** | 0.001 |

Table S12. Model summaries for generalized linear models of non-native ambrosia beetle abundance within deadwood objects. Each model excludes a portion of the dataset originating from a unique study (Table 1 in the Main Text), had random effects for study and tree family, and had offsets for wood volume and total number of ambrosia beetles in the object. Z-values in bold are significant (*p*-value < 0.05).

| Response: Non-native ambrosia beetle abundance | | | | | |
| --- | --- | --- | --- | --- | --- |
| Excluded study | Variables | Estimate | SE | z-value | p-value |
| Excluding Ulyshen and Hanula (2009) | (Intercept) | 5.016 | 0.754 | **6.657** | 2.80×10^-11^ |
|  | log(1 + Native abundance) | -0.298 | 0.087 | **-3.417** | 0.001 |
|  | Origin (native) | -0.164 | 0.223 | -0.734 | 0.463 |
|  | Type (broadleaf) | -1.072 | 0.616 | -1.740 | 0.082 |
|  | Time (months) | -0.006 | 0.038 | -0.148 | 0.882 |
| Excluding Ulyshen et al. (2018) | (Intercept) | 3.897 | 0.803 | **4.855** | 1.20×10^-06^ |
|  | log(1 + Native abundance) | -0.312 | 0.215 | -1.448 | 0.148 |
|  | Origin (native) | 0.182 | 0.726 | 0.251 | 0.802 |
|  | Type (broadleaf) | 1.192 | 0.759 | 1.570 | 0.116 |
|  | Time (months) | -0.302 | 0.105 | **-2.891** | 0.004 |
| Excluding Ulyshen et al. (2024) | (Intercept) | -16.705 | 5200.987 | -0.003 | 0.997 |
|  | log(1 + Native abundance) | -0.372 | 0.099 | **-3.758** | 1.72×10^-04^ |
|  | Origin (native) | -0.542 | 0.473 | -1.147 | 0.251 |
|  | Type (broadleaf) | 21.817 | 5200.987 | 0.004 | 0.997 |
|  | Time (months) | -0.185 | 0.044 | **-4.185** | 2.85×10^-05^ |
| Excluding present study | (Intercept) | 1.901 | 1.568 | 1.212 | 0.225 |
|  | log(1 + Native abundance) | -0.703 | 0.311 | **-2.257** | 0.024 |
|  | Origin (native) | 1.438 | 1.490 | 0.966 | 0.334 |
|  | Type (broadleaf) | 2.478 | 1.424 | **1.740** | 0.082 |
|  | Time (months) | -0.166 | 0.110 | -1.505 | 0.132 |

Table S13. Model summaries for generalized linear models of the probability that an emerging ambrosia beetle is native. Each model excludes a portion of the dataset originating from a unique study (Table 1 in the Main Text) and had random effects for study and tree family. Z-values in bold are significant (*p*-value < 0.05).

| Response: Probability of an emerging beetle being native | | | | | |
| --- | --- | --- | --- | --- | --- |
| Excluded study | Variables | Estimate | SE | z-value | p-value |
| Excluding Ulyshen and Hanula (2009) | (Intercept) | -2.417 | 3.228 | -0.749 | 0.454 |
|  | Origin (native) | 1.295 | 0.530 | **2.444** | 0.015 |
|  | Type (broadleaf) | 3.210 | 3.197 | 1.004 | 0.315 |
|  | Wood volume (m^3^) | 31.213 | 19.724 | 1.583 | 0.114 |
|  | Time (months) | -0.766 | 0.081 | **-9.405** | 5.19×10^-21^ |
| Excluding Ulyshen et al. (2018) | (Intercept) | 11.812 | 8.193 | 1.442 | 0.149 |
|  | Origin (native) | 0.814 | 0.543 | 1.499 | 0.134 |
|  | Type (broadleaf) | -1.381 | 1.795 | -0.769 | 0.442 |
|  | Wood volume (m^3^) | 64.672 | 4.324 | **14.957** | 1.40×10^-50^ |
|  | Time (months) | -2.411 | 0.580 | **-4.159** | 3.19×10^-05^ |
| Excluding Ulyshen et al. (2024) | (Intercept) | 19.739 | 4016.821 | 0.005 | 0.996 |
|  | Origin (native) | 1.244 | 0.519 | **2.397** | 0.017 |
|  | Type (broadleaf) | -19.254 | 4016.821 | -0.005 | 0.996 |
|  | Wood volume (m^3^) | 62.051 | 4.124 | **15.045** | 3.70×10^-51^ |
|  | Time (months) | -0.410 | 0.038 | **-10.717** | 8.43×10^-27^ |
| Excluding present study | (Intercept) | 4.270 | 7.346 | 0.581 | 0.561 |
|  | Origin (native) | 0.496 | 4.544 | 0.109 | 0.913 |
|  | Type (broadleaf) | -4.107 | 6.163 | -0.666 | 0.505 |
|  | Wood volume (m^3^) | 61.598 | 4.133 | **14.904** | 3.08×10^-50^ |
|  | Time (months) | -0.490 | 0.040 | **-12.136** | 6.77×10^-34^ |
